# Naoxintong capsule delay the progression of diabetic kidney disease: A real-world cohort study

**DOI:** 10.3389/fendo.2022.1037564

**Published:** 2022-11-03

**Authors:** Yuqing Zhang, Yuehong Zhang, Cunqing Yang, Yingying Duan, Linlin Jiang, De Jin, Fengmei Lian, Xiaolin Tong

**Affiliations:** ^1^ Endocrinology Department, Guang’anmen Hospital, China Academy of Chinese Medical Sciences, Beijing, China; ^2^ Endocrinology Department, Guang’anmen Hospital, Beijing University of Chinese Medicine, Beijing, China; ^3^ Department of Nephrology, Hangzhou Hospital of Traditional Chinese Medicine, Hangzhou, Zhejiang, China; ^4^ Institute of Metabolic Diseases, Guang’anmen Hospital, China Academy of Chinese Medical Sciences, Beijing, China

**Keywords:** Naoxintong capsule, diabetic kidney disease, retrospective cohort study, propensity score matching, real-world study

## Abstract

**Introduction:**

Diabetic kidney disease (DKD) is a severe and growing health problem, associated with a worse prognosis and higher overall mortality rates than non-diabetic renal disease. Chinese herbs possess promising clinical benefits in alleviating the progression of DKD due to their multi-target effect. This real-world retrospective cohort trial aimed to investigate the efficacy and safety of Naoxintong (NXT) capsules in the treatment of DKD. Our study is the first real-world study (RWS) of NXT in the treatment of DKD based on a large database, providing a basis for clinical application and promotion.

**Methods:**

The data was collected from Tianjin Healthcare and Medical Big Data Platform. Patients with DKD were enrolled from January 1, 2011, to March 31, 2021. NXT administration was defined as the exposure. The primary outcome was the change in estimated glomerular filtration rate (eGFR). We employed the propensity score matching (PSM) method to deal with confounding factors.

**Results:**

A total of 1,798 patients were enrolled after PSM, including 899 NXT users (exposed group) and 899 non-users (control group). The eGFR changes from baseline to the end of the study were significantly different in the exposed group compared to the control group (-1.46 ± 21.94 vs -5.82 ± 19.8 mL/(min·1.73m^2^), P< 0.01). Patients in the NXT group had a lower risk of composite renal outcome event (HR, 0.71; 95%CI, 0.55 to 0.92; P = 0.009) and deterioration of renal function (HR, 0.74; 95% CI, 0.56 to 0.99; P = 0.039).

**Conclusion:**

NXT can significantly slow the decline of eGFR and reduce the risk of renal outcomes. However, large cohort studies and RCTs are needed to further confirm our results.

## Introduction

Diabetic kidney disease (DKD) is a severe and growing health problem globally, which is reported to be the leading cause of the end-stage renal disease (ESRD) (1). There is accumulating evidence that DKD is associated with a worse prognosis and higher overall mortality rates than non-diabetic renal disease (2). Efforts to reduce ESRD prevalence and burden, therefore, include the prevention of DKD before its occurrence and the delay of progression of established DKD. Recent guidelines on the treatment of DKD recommended that lifestyle interventions and the blocking of the renin-angiotensin–aldosterone system (RAAS) should be considered for individuals with DKD (3). Despite the certain effects these recommendations achieved, many individuals still suffer glomerular filtration rate (GFR) decline and renal damage due to the lack of satisfactory therapeutic strategies (4).

Traditional Chinese Medicine (TCM) has a long history of clinical practice and is becoming a promising option worldwide as complementary medicine (5). TCM therapies, especially Chinese herbs, possess promising clinical benefits in alleviating the progression of DKD due to their multi-target effect (6). Naoxintong (NXT) capsule is one of the classic prescription drugs for treating cardiovascular disease caused by Qi stagnation and blood stasis (7). The formula has been reported to have a renal protective effect and be effective in ameliorating glucose metabolism and delaying DKD progression (8, 9). However, to our knowledge, NXT capsule has never been investigated in DKD patients in the setting of a clinical trial. The prior studies were limited to the empirical summaries of physicians and had a small sample size. Therefore, we hypothesize that NXT capsule may be a safe and effective treatment for DKD. We designed a retrospective observational cohort study to evaluate the efficacy and safety of NXT in patients with DKD. Our study is the first real-world study (RWS) of NXT in the treatment of DKD based on a large database, providing a basis for clinical application and promotion.

## Methods

### Data collection

The present retrospective observational cohort study was conducted using data from Tianjin Healthcare and Medical Big Data Platform. This platform contains information from 42 tertiary hospitals and 40 secondary hospitals, supplemented by data from 274 primary and community hospitals, covering 17,420,097 patients’ medical records in Tianjin, China. Patient data were retrieved from the electronic medical record, Hospital Information System (HIS), Laboratory Information System (LIS), and the home page of the medical record, including the date of birth, sex, BMI, diagnostic codes, course of the disease, medication, and laboratory indices. The data were extracted by two researchers to avoid bias and were validated by a third researcher to ensure their accuracy.

This study was approved by the ethics committee of Guang’anmen Hospital, China Academy of Chinese Medical Sciences on November 23, 2020 (2020-056-KY). Since the study was designed retrospectively, informed consent was not required. All clinical studies were conducted in accordance with the Helsinki Declaration.

### Study design and participants

Patients with confirmed DKD from 82 hospitals were enrolled from January 1, 2011, to March 31, 2021. The diagnostic criteria of DKD were according to the Kidney Disease Outcomes Quality Initiative (KDOQI) clinical practice guidelines (10). Key inclusion criteria included estimated glomerular filtration rate<90 mL/(min·1.73m^2^) and sufficient serum creatinine records or endpoint events. Patients with a severe lack of clinical diagnosis and treatment data were excluded. The inclusion and exclusion criteria are as follows:

#### Inclusion criteria

(1) Patients in the database who met any of the following diagnoses:
a. Diagnostic names included “diabetic nephropathy”, “diabetic kidney disease”, “diabetes with renal complications” or disease ICD is “E10.2”, “E11.2”, “E12.2”, “E13.2”, “E14.22”;
b. Patients who met both of the following diagnoses: ①Diagnostic names included “diabetes”, “hyperglycemia”, or disease ICD is “E14.901”, “R73.901”; ②Diagnostic names included “arteriosclerotic nephropathy”, “hypertensive nephropathy”, “renal disease”, “nephropathy”, or disease ICD is “I12.903”, “N04.901”, “N28.901”, “I12.901”;
(2) Baseline glomerular filtration rate was less than 90 mL/(min·1.73m^2^);
(3) The visit time was from January 1, 2011 to March 31, 2021;
(4) Two serum creatinine tests were recorded or at least one serum creatinine test with one endpoint event.

#### Exclusion criteria

1. Patients with serious lack of clinical diagnosis and treatment data;
2. Patients with NXT medication interval of more than 1 year.

**Figure 1:**
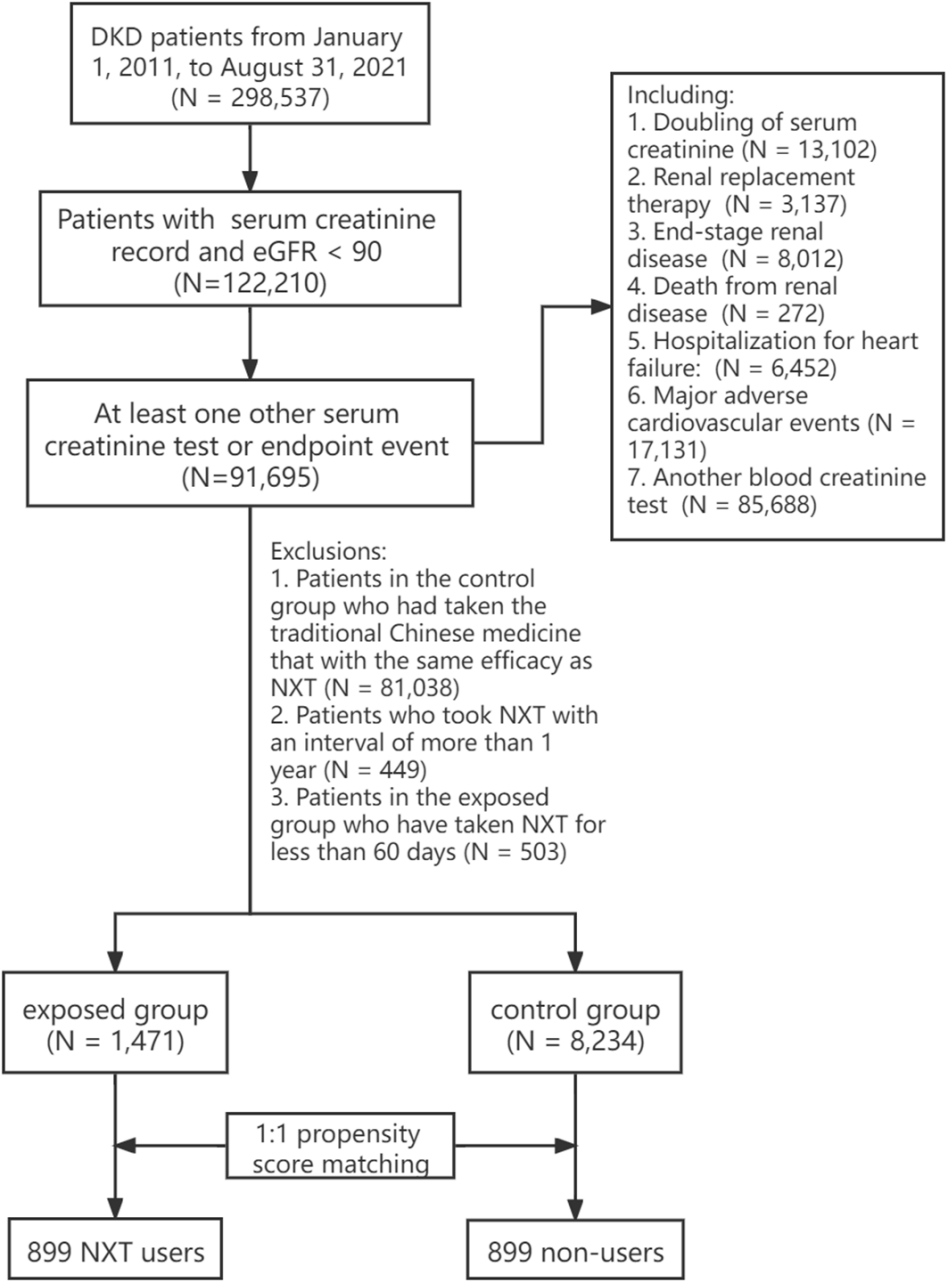
Workflow of the retrospective cohort study.

The patients were divided into two groups according to the NXT prescription. The exposed group included patients who had taken NXT for more than 60 days, and it was further divided into high, medium, and low exposed groups based on the duration of administration. The control group included patients who were diagnosed with DKD but did not take NXT (including capsule, decoction, granules, and traditional Chinese medicine that with the same efficacy as NXT). Since the data in this study are real-world data, propensity score matching (PSM) was conducted to reduce the interference of confounding factors. The final study cohort comprised 1,798 DKD patients, with 899 patients in each group (**Figure 1**). Details on the study design and screening process can be found in **Supplement 1**.

### Outcomes

The primary outcome was the change in estimated glomerular filtration rate. The eGFR was calculated by CKD-EPI equation using gender, age, and serum creatinine. The formula is as follows:


$$\text{Female}\leq 62\left(\leq 0.7\right)\mu \text{mol}/\text{L}\left(\text{mg}/\text{dL}\right)\;\text{eGFR}［\text{mL}/\left(\text{min}\cdot 1.73{\text{m}}^{2}\right)］=144\times {\left(\text{Scr}/0.7\right)}^{-0.329}\times {\left(0.993\right)}^{\text{age}}＞62\left(>0.7\right)\mu \text{mol}/\text{L}\left(\text{mg}/\text{dL}\right)\;\;\text{eGFR}［\text{mL}/\left(\text{min}\cdot 1.73{\text{m}}^{2}\right)］=144\times {\left(\text{Scr}/0.7\right)}^{-1.209}\times {\left(0.993\right)}^{\text{age}}$$

$$\text{Male}\;\leq 80\left(\leq 0.9\right)\mu \text{mol}/\text{L}\left(\text{mg}/\text{dL}\right)\text{ eGFR}［\text{mL}/\left(\text{min}\cdot 1.73{\text{m}}^{2}\right)］=141\times {\left(\text{Scr}/0.9\right)}^{-0.411}\times {\left(0.993\right)}^{\text{age}}\;＞80\left(>0.9\right)\mu \text{mol}/L\left(\text{mg}/\text{dL}\right)\text{ eGFR}［\text{mL}/\left(\text{min}\cdot 1.73{\text{m}}^{2}\right)］=141\times {\left(\text{Scr}/0.9\right)}^{-1.209}\times {\left(0.993\right)}^{\text{age}}$$

The secondary outcomes included kidney composite endpoint (end-stage renal disease (11), renal replacement therapy, doubling of serum creatinine (12), death due to kidney disease), 50% decrease in eGFR, major adverse cardiovascular events (MACE) (cardiovascular death, non-fatal myocardial infarction or non-fatal stroke), hospitalization for heart failure, and the change of CKD stages.

The stage of CKD refers to the 2021 definition and staging criteria of CKD proposed by Kidney Disease: Improving Global Outcomes (KDIGO). Safety indicators included blood routine, serum electrolyte, and liver function, which were assessed by calculating the difference between the endpoint and baseline.

### Statistical analysis

For quantitative data, descriptive statistical analysis was carried out by the number of cases (N), Mean, standard deviation (Std), minimum (Min), Maximum (Max), median (Med), upper quartile (Q1), and lower quartile (Q3). For qualitative data, descriptive statistical analysis was performed using frequency tables, percentages, or constituent ratios. Differences between continuous normally-distributed variables were tested with the Student t-test; non-normally distributed variables were compared by the rank-sum test (13). Categorical variables were compared with the chi-squared test (14). For survival data or time-to-event data, the Kaplan-Meier survival analysis model was used to calculate the average survival time. Survival curves (Kaplan-Meier curves) were drawn to describe the survival status of patients in each group, with HRs and 95% CIs estimated using a Cox proportional hazards model.

To eliminate the influence of baseline covariates, we employed the PSM method to deal with potential confounding factors (15, 16). Literature search and stepwise logistic regression were used to select the confounders with relatively large impact on the results. Finally, the following confounding factors were included in the PSM model:

Age, gender, smoking history, alcohol consumption history, BMI, blood pressure classification, observation days, baseline eGFR, baseline medication (Insulin and its analogs, glinides, sulfonylurea, biguanides, thiazide diuretics, β-blockers, ACEI/ARB), underlying diseases (hypertension, cardiovascular and cerebrovascular diseases, chronic respiratory diseases, metabolic diseases, urinary system diseases, anemia).

The propensity score matching method was carried out by the MatchIt package of R software (17). According to whether NXT was taken or not, the above factors were used as covariables to construct the propensity score (PS) by logistic regression analysis. We used the 1:1 nearest neighbor matching method to match the propensity scores, and the maximum caliper width was set to 0.25 of the PS standard deviation (18). After the completion of PSM, the standardized mean differences were used to assess the balance of covariates before and after matching, with an absolute value less than 0.1 indicating acceptable balance and reasonable control of confounding factors (19).

For all tests, significance was defined as P ≤ 0.05. SPSS 22.0 and R 3.6.2 software were employed for statistical analysis (20, 21).

## Results

### Demographic and clinical characteristics

A total of 9,705 patients were enrolled in the final cohort, with 1,471 (15.2%) patients in exposed group and 8,234 (84.8%) patients in control group (**Figure 1**). Before PSM, NXT users had lower eGFR (72.97 ± 17.53 vs 69.61 ± 21.10 mL/(min·1.73m^2^)), longer observation days (1636.37 days vs 926.40 days), and a higher proportion with comorbidities and concomitant medication. We found a significantly higher percentage of patients in the exposed group had a history of hypertension (99.3% vs 69.8%, P< 0.001), cardio-cerebrovascular diseases (99.8% vs 73.9%, P< 0.001), and metabolic diseases (77.8% vs 43.6%, P< 0.001) compared with the control group. And the proportion of patients using Insulin and its analogs (77% vs 46.5%, P< 0.001), glinides (43.8% vs 16.7%, P< 0.001), sulfonylurea (30.6% vs 15.7%, P< 0.001), biguanides (57.0% vs 30.7%, P< 0.001), thiazide diuretics (9.9% vs 1.4%, P< 0.001), β-blockers (31.3% vs 13.9%, P< 0.001), ACEI (69.8% vs 13.6%, P< 0.001), and ARB (18.6% vs 4.1%, P< 0.001) was significantly higher in the exposed group than in the control group.

Considering the interference of confounding factors, the PSM method was employed to balance the baseline covariates (22). Of 8,234 patients eligible for analysis in the control group, 899 were matched to a patient from NXT exposed group. After 1:1 propensity score–matching, all variables were balanced between the exposed group and control group (**Table 1**). The standardized mean differences were calculated to assess the balance in the PS model and were<0.1. In the exposed group, the median time of NXT taken was 224 days. Estimated glomerular filtration rate in exposed group and analysis-eligible control group were 72.18 ± 18.49 mL/(min·1.73m^2^) and 71.99 ± 18.98mL/(min·1.73m^2^), respectively. In subgroup analysis, the high, middle, and low exposed groups contained 306, 233, and 360 patients, respectively. All variables were balanced between the three subgroups and their respective matched control groups. There were no significant differences in baseline eGFR levels among the 3 subgroups. Details on baseline characteristics of each subgroup before and after PSM could be found in **Supplement 2 (Table I-IV)**.

**Table 1:** The baseline characteristic before and after propensity score matching.

Group	Before PSM				After PSM			
	Exposed group (1471)	Control group (8234)	P Value	Std. Mean Diff.	Exposed group (899)	Control group (899)	P Value	Std. Mean Diff.
Age (mean (SD))	66.71 (9.18)	63.37 (11.00)	<0.001	0.3643	66.17 (9.17)	66.13 (10.38)	0.923	0.0049
Male (%)	936 (63.6)	4629 (56.2)	<0.001	0.1541	555 (61.7)	531 (59.1)	0.267	0.0549
Smoking history (%)	364 (24.7)	879 (10.7)	<0.001	0.326	163 (18.1)	177 (19.7)	0.668	-0.0404
Drinking history (%)	272 (18.5)	721 (8.8)	<0.001	0.2507	135 (15.0)	137 (15.2)	0.971	-0.0062
BMI (%)			0.005				0.99	
obese (BMI≥25)	194 (13.2)	1168 (14.2)		-0.0295	128 (14.2)	128 (14.2)		0
overweight (23≥BMI<25)	366 (24.9)	2003 (24.3)		0.0128	211 (23.5)	211 (23.5)		0
normal (18.5≥BMI<23)	311 (21.1)	1424 (17.3)		0.0942	182 (20.2)	177 (19.7)		0.0138
underweight (BMI<18.5)	5 (0.3)	26 (0.3)		0.0041	2 (0.2)	3 (0.3)		-0.0236
Blood pressure classification (%)			<0.001				0.92	
Grade 1 hypertension	208 (14.1)	835 (10.1)		0.1148	113 (12.6)	116 (12.9)		-0.0101
Grade 2 hypertension	80 (5.4)	338 (4.1)		0.0588	47 (5.2)	53 (5.9)		-0.03
Grade 3 hypertension	23 (1.6)	123 (1.5)		0.0056	14 (1.6)	10 (1.1)		0.0359
High normal	668 (45.4)	3426 (41.6)		0.0764	395 (43.9)	383 (42.6)		0.0269
Normal	57 (3.9)	357 (4.3)		-0.0239	37 (4.1)	35 (3.9)		0.0112
Baseline eGFR (mean (SD))	72.97 (17.53)	69.61 (21.10)	<0.001	0.1917	72.18 (18.49)	71.99 (18.98)	0.825	0.0106
Observation days (mean (SD))	1636.37 (1037.36)	926.40 (1004.00)	<0.001	0.6844	1373.86 (997.67)	1392.85 (1029.45)	0.691	-0.019
Baseline medication								
Insulin and its analogs (%)	1132 (77.0)	3826 (46.5)	<0.001	0.724	608 (67.6)	614 (68.3)	0.8	-0.0143
Glinides (%)	645 (43.8)	1374 (16.7)	<0.001	0.5474	293 (32.6)	301 (33.5)	0.726	-0.019
Sulfonylurea (%)	450 (30.6)	1294 (15.7)	<0.001	0.3228	212 (23.6)	213 (23.7)	1	-0.0026
Biguanides (%)	839 (57.0)	2526 (30.7)	<0.001	0.5325	426 (47.4)	421 (46.8)	0.85	0.0111
Thiazide diuretics (%)	145 (9.9)	117 (1.4)	<0.001	0.283	47 (5.2)	50 (5.6)	0.835	-0.015
β-blockers (%)	460 (31.3)	1143 (13.9)	<0.001	0.3751	256 (28.5)	263 (29.3)	0.755	-0.0173
ACEI (%)	1027 (69.8)	1122 (13.6)	<0.001	1.224	484 (53.8)	483 (53.7)	1	0.0022
ARB (%)	273 (18.6)	337 (4.1)	<0.001	0.3721	98 (10.9)	97 (10.8)	1	0.0036
Underlying diseases								
Hypertension (%)	1461 (99.3)	5748 (69.8)	<0.001	3.5916	889 (98.9)	888 (98.8)	1	0.0106
Cardio-cerebrovascular diseases (%)	1468 (99.8)	6085 (73.9)	<0.001	5.7399	896 (99.7)	893 (99.3)	0.504	0.0579
Chronic respiratory diseases (%)	385 (26.2)	880 (10.7)	<0.001	0.3523	189 (21.0)	194 (21.6)	0.818	-0.0136
Metabolic diseases (%)	1145 (77.8)	3593 (43.6)	<0.001	0.8235	607 (67.5)	610 (67.9)	0.92	-0.0071
Urinary system diseases (%)	379 (25.8)	1284 (15.6)	<0.001	0.2326	207 (23.0)	198 (22.0)	0.652	0.0238
Anemia (%)	316 (21.5)	1469 (17.8)	<0.001	0.0887	184 (20.5)	197 (21.9)	0.489	-0.0358

SD, Standard Deviation; ACEI, angiotensin-converting enzyme inhibitor; ARB, angiotensin II receptor blocker.

### Primary outcome

**Table 2** presented the changes in eGFR at the endpoint compared to the baseline. The mean baseline eGFR is 72.18 ± 18.49 mL/(min·1.73m^2^) in exposed group and 71.99 ± 18.98mL/(min·1.73m^2^) in control group, with no statistical difference between the two groups (P = 0.96). After NXT administration, the exposed group had higher eGFR (70.73 ± 27.68 vs 66.17 ± 26.43 mL/(min·1.73m^2^)) compared to non-exposed group, with p-value< 0.01. The eGFR changes from baseline to the end of the study were significantly different in the exposed group compared to the control group (-1.46 ± 21.94 vs -5.82 ± 19.8 mL/(min·1.73m^2^), P< 0.01). In the exposed group, the overall rate of decline in eGFR was 0.07 ± 13.42mL/min/1.73 m^2^ per year, compared with 1.91 ± 19.34 mL/min/1.73 m^2^ per year in the control group (P< 0.01).

**Table 2:** The change of eGFR before and after study.

	Exposed group	Control group	W statistic	P-Value
Baseline eGFR	72.18±18.49	71.99±18.98	403532	0.958846
Endpoint eGFR	70.73±27.68	66.17±26.43	351593	0.000002
eGFR change	-1.46±21.94	-5.82±19.8	350706	0.000001

**Figure 2:**
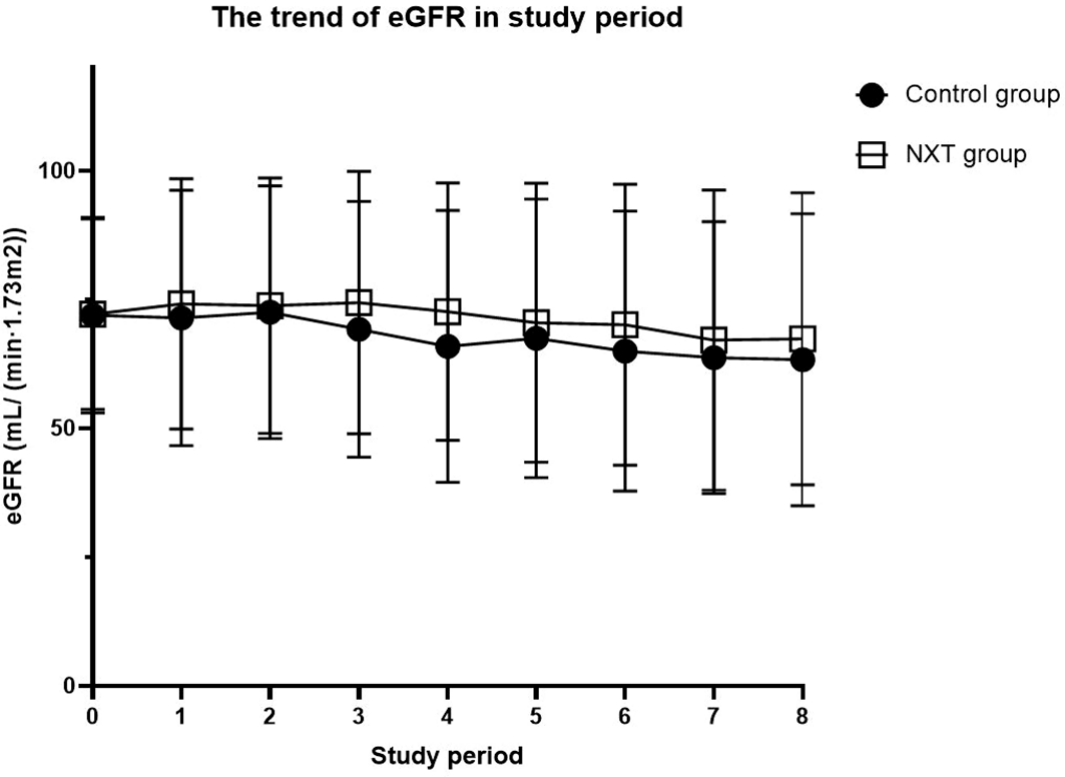
The trend of eGFR in study period. 1 study period = 180 days.

The trend of eGFR in the study period was presented in **Table 3** and **Figure 2**. Starting from the baseline, we defined a period of 180 days as a follow-up time point. The mean eGFR was calculated for each period. From the overall trend, the eGFR in the NXT group improved to a certain extent, with P values of 0.0003, 0.0005, and 0.023 in the third, fourth and sixth study periods, respectively.

**Table 3:** The trend of eGFR in study period.

Study period	Control group	NXT group	number of patients in control group	number of patients in NXT	W statistic	P-Value
baseline	71.99 ± 18.98	72.18 ± 18.49	899	899	403532	0.958846
1	71.45 ± 24.8	74.15 ± 24.29	447	357	74341.5	0.095922
2	72.54 ± 24.56	73.83 ± 24.76	413	373	72696	0.173313
3	69.24 ± 24.85	74.43 ± 25.49	340	332	47436	0.000346
4	65.92 ± 26.4	72.67 ± 25.01	299	323	40433	0.000452
5	67.5 ± 27.04	70.53 ± 27.11	216	309	30360.5	0.078346
6	64.98 ± 27.18	70.13 ± 27.3	170	281	20825.5	0.02258
7	63.71 ± 26.42	67.13 ± 29.13	148	228	14422.5	0.061622
8	63.35 ± 28.37	67.38 ± 28.37	123	194	10868	0.181503

1 study period = 180 days.

### Secondary outcome

**Figure 3:**
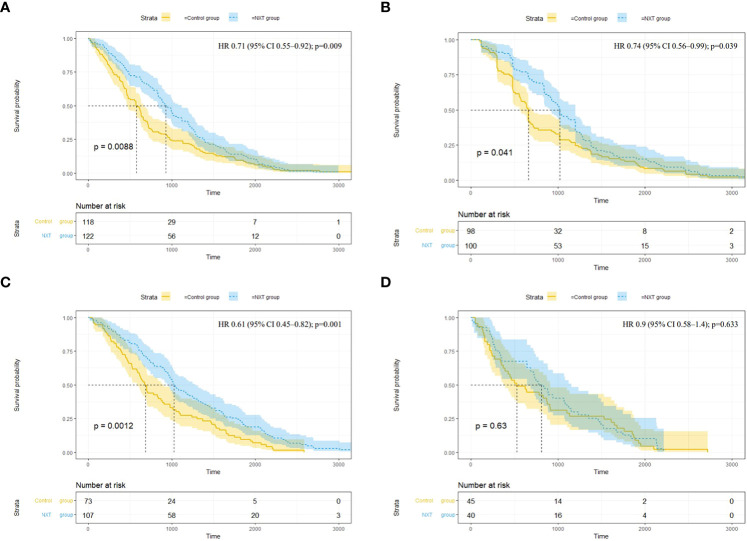
Survival analysis of endpoint events. **(A)** Composite renal outcome (end-stage renal disease11, renal replacement therapy, doubling of serum creatinine, death due to kidney disease); **(B)** 50% decrease in eGFR; **(C)** Major adverse cardiovascular events (MACE) (cardiovascular death, non-fatal myocardial infarction or non-fatal stroke); **(D)** Hospitalization for heart failure. HR, hazard ratio; CI, confidence interval; Time (days).

Significant benefit in the time to the first occurrence of composite renal outcome was observed in the NXT group (hazard ratio [HR], 0.71; 95% confidence interval [CI], 0.55 to 0.92; P = 0.009) (**Figure 3A**). The median occurrence time was 929.5 (95% CI, 838 to 1118) days in the exposed group versus 575.5 (95% CI, 463 to 662) days in the control group. There were 100 patients presented the outcome of deterioration of renal function in the exposed group and 98 in the control group (HR, 0.74; 95% CI, 0.56 to 0.99; P = 0.039) (**Figure 3B**). The median occurrence time was significantly longer in the exposed group (1020 days; 95% CI, 919 to 1200 versus 660 days; 95% CI, 568 to 712). Longer survival time of MACE was observed in NXT group (HR, 0.61; 95% CI, 0.45 to 0.82; P = 0.001) (**Figure 3C**). However, the incidence of MACE was higher in the exposed group (8.1%) than in the control group (11.9%). There was no significant difference in the time to hospitalization for heart failure (P = 0.63) (**Figure 3D**).

In terms of the change of CKD stages, more patients in the exposed group were in CKD stage 1 at the endpoint (269 vs 159) compared with the control group, exhibiting a noticeable improvement in eGFR. (**Table 4**). The overall improvement proportion of CKD stage was 34.04% in the exposed group and 22.36% in the control group, while the overall progression proportion of CKD stage was 21.25% in the exposed group and 24.92% in the control group.

**Table 4:** The change of CKD stages.

a. The change of CKD stages in exposed group						
Endpoint Baseline	CKD1	CKD2	CKD3a	CKD3b	CKD4	CKD5
CKD2	249	346	52	34	30	11
CKD3a	13	23	25	16	10	5
CKD3b	5	4	4	8	8	12
CKD4	2	1	1	2	5	13
CKD5	0	0	0	0	2	18
Total	269	374	82	60	55	59
b. The change of CKD stages in control group						
Endpoint Baseline	CKD1	CKD2	CKD3a	CKD3b	CKD4	CKD5
CKD2	156	399	93	32	25	17
CKD3a	3	34	28	19	13	5
CKD3b	0	1	4	12	5	6
CKD4	0	1	0	2	5	9
CKD5	0	0	0	0	0	30
Total	159	435	125	65	48	67
c. The proportion of changes in CKD stages						
		Improvement	maintenance	progression		
Exposed group	Total	306 (34.04%)	402 (44.72%)	191 (21.25%)		
	CKD2-CKD3a	285 (35.01%)	371 (45.58%)	158 (19.41%)		
	CKD3b-CKD5	21 (24.7%)	31 (36.47%)	33 (38.82%)		
Control group	Total	201 (22.36%)	474 (52.73%)	224 (24.92%)		
	CKD2-CKD3a	193 (23.42%)	427 (51.82%)	204 (24.76%)		
	CKD3b-CKD5	8 (10.67%)	47 (62.67%)	20 (26.67%)		

The difference in safety indices between baseline and the end of the study is shown in **Table 5**. NXT capsule did not cause any abnormality of safety indicators including blood routine, serum electrolyte, and liver function (P>0.05).

**Table 5:** The difference of safety indices before and after treatment.

	Control group	NXT group	Number of patients in control group	Number of patients in NXT group	W statistic	P-Value
RBCs	-0.17 ± 0.56	-0.15 ± 0.68	605	664	192327.5	0.190696
WBCs	0.36 ± 2.91	0.46 ± 3.57	605	665	200460.5	0.914422
PLT	-4.67 ± 53.16	1.79 ± 64.67	604	664	191494	0.165402
HbA1c	-5.01 ± 17.24	-5.26 ± 20.98	559	541	148217	0.570007
K+	-0.02 ± 0.6	0.01 ± 0.59	376	441	80694.5	0.510382
ALT	4.76 ± 73.76	4.16 ± 75.86	502	520	127785	0.562135
AST	10.2 ± 64.83	16.96 ± 174.09	336	346	53247	0.057786
ALP	1.92 ± 54.07	2.3 ± 53.07	484	534	124114.5	0.275087
TBIL	0.68 ± 8.98	0.07 ± 8.15	512	552	141388	0.987972
TP	5.59 ± 66.58	5.27 ± 52.83	451	485	103663	0.167512

RBCs, red blood cells; WBCs, white blood cells; PLT, platelets; HbA1c, Hemoglobin A1c; K+, potassium; ALT, alanine aminotransferase; AST, aspartate aminotransferase; ALP, Alkaline phosphatase; TBIL, total bilirubin; TP, transpeptidase.

## Discussion

DKD has emerged as a worldwide medical catastrophe, bringing tremendous pressure on medical resources and the economy. According to data from the International Diabetes Federation (IDF), by the end of 2021, the number of diagnosed diabetes mellitus (DM) reached 537 million globally (23), which has a great impact on the prevalence rate of DKD. Much effort has been made to slow the reduction of GFR and maintain renal function, including glycemic control, blood pressure regulation, and lipid lowering (24). However, the long‐term impact of these strategies is still not optimistic (25).

Based on the current treatment status, TCM treatment of diabetic kidney disease needs to be paid more attention. Our study is the first real-world study (RWS) of TCM treatment of DKD based on a large electronic information system. The reason we conducted RWS was that it is designed to evaluate the outcomes of all interventions in a real clinical setting to achieve results that are more closely related to clinical reality. Moreover, although Naoxintong capsule has been commonly used clinically for the treatment of DKD, it has only been approved for the treatment of cardiovascular and cerebrovascular diseases, which makes it impossible to conduct RCTs related to DKD. RWS could provide basis for NXT off-label drug use, further expand drug indications, and lay a foundation for follow-up randomized controlled studies. Our study demonstrated that TCM was efficacious in slowing eGFR decline and reducing the progression of early-stage DKD to ESRD. Thus, TCM can be an alternative treatment in addition to glucose-lowering, antihypertensive, and lipid-lowering therapies for the treatment of DKD patients.

In this retrospective cohort study, patients with DKD who used NXT capsules had less eGFR decline (1.46 ± 21.94 vs 5.82 ± 19.8 mL/(min·1.73m^2^)) than those who did not take NXT. Although the incidence rates were similar, patients in the NXT group had a lower risk of composite renal outcome events (end-stage renal disease, renal replacement therapy, doubling of serum creatinine, death due to kidney disease), and deterioration of renal function (50% decrease in eGFR). These results suggest that NXT capsules may be an effective therapy for kidney protection in DKD patients. A longer survival time of MACE was also observed in patients who used NXT (HR, 0.61; 95% CI, 0.45 to 0.82; P = 0.001). However, we found a higher incidence of MACE in the NXT group compared with the control group (11.9% vs 8.1%). This may be due to the fact that clinicians prescribe NXT capsules mainly for patients with cardiovascular diseases (CVD). Therefore, patients who were administrated NXT were more likely to have worse cardiovascular situations. Our study focuses more on the therapeutic effect of NXT on DKD, confounding factors related to cardiovascular disease may not be well balanced.

Moreover, NXT administration was significantly associated with the improvement of CKD stages both in the subgroup of patients with CKD stage 2-3a and CKD stage 3b-5. The number of patients with improved CKD stage in the NXT group and the control group was 306 and 201 (34.04% vs 22.36%), respectively. In terms of CKD progression, NXT showed clinical benefits in patients with CKD stage 2-3a (19.41% vs 24.76%). While in patients with CKD stage 3b-5, NXT capsules did not achieve ideal results (38.82% vs 26.67%). This suggests that NXT may have greater benefit in improving renal function in early-stage DKD patients. For patients with CKD stage 3b-5, the proportion of progression and improvement was higher in the NXT group than in the control group. At the same time, due to the relatively small sample size of late-stage DKD patients, there may be some bias in the results.

In addition, the patients in the exposed group were divided into three subgroups according to exposure time, and the average duration of medication in the high, medium, and low exposed groups was 700.28 days, 253.20 days, and 106.82 days, respectively (**Supplement 2 Table IV**). we observed that NXT administration ameliorated the eGFR decline and improved the CKD stages, irrespective of the level of exposure to NXT (**Supplement 2 Table V-VI**).

NXT capsule was developed from the classical formula “Bu-Yang-Huan-Wu-Tang (BYHWT)”, which has been approved by the China Food and Drug Administration (CFDA) for the treatment of cardiovascular disease (7). Previous studies have shown that NXT takes a variety of protective effects on CVD including coronary artery disease, myocardial infarction, atherosclerosis, acute coronary syndrome, ischemic stroke, and so on (26–29). In addition, researchers found that NXT may also have renoprotective effects by improving insulin sensitivity and regulating glucose and lipid metabolism. Yang et al. found that NXT protects db/db diabetic mice from DN by reducing renal inflammation and podocyte injury (8). Moreover, NXT decreased the deposition of extracellular matrix proteins by increasing the expression of MMP2/9 through inhibition of the TGF-β/Smad pathway and CTGF expression (9). Yan et al. demonstrated that NXT exerted therapeutic efficacy against diabetes and its complications by improving insulin sensitivity, glucose metabolism, and energy expenditure (30). However, the above-mentioned studies mainly focused on animal experiments, without the support of clinical studies. Due to the off-label use of NXT in the treatment of DKD, randomized controlled trials (RCTs) cannot be conducted. Therefore, we conducted a retrospective study to explore the therapeutic effect of NXT, providing scientific evidence and a research basis for expanding the indications of NXT capsules. The research database we selected covers all hospitals in Tianjin area, with wide data coverage and sufficient number of patients. The patient data captured in this database are representative, providing reliable resources for us to carry out the study. Our study showed that NXT is effective in improving eGFR in DKD patients, especially in patients with early-stage DKD. NXT also had a beneficial effect on the risk of renal events, as demonstrated by the increase in survival time of the renal composite outcome.

NXT contains sixteen Chinese materia medica: Radix Astragali (Huangqi), Radix Paeoniae Rubra (Chishao), Salviae miltiorrhizae Radix et Rhizoma (Danshen), Radix Angelicae Sinensis (Danggui wei), Chuanxiong Rhizoma (Chuanxiong), Persicae Semen (Taoren), Carthami Flos (Honghua), Olibanum (Ruxiang), Myrrha (Moyao), Spatholobi Stem (Jixueteng), Cinnamomi Ranulus (Guizhi), Achyranthis bidentatae Radix (Niuxi), Scorpio (Quanxie), Pheretima (Dilong), Mori Ramulus (Sangzhi), and Hirudo (Shuizhi) (7). In TCM theory, these prescriptions can tonify qi, activate blood circulation, remove stasis, and dredge collaterals. Thus, the indications of NXT coincide with the main pathogenesis of DKD, which is characterized by the presence of qi deficiency, blood stasis, and turbidity. Studies have shown that many components of NXT possess anti-inflammation properties and could improve renal function (8). The main identified components in the NXT capsule include ferulic acid, paeoniflorin, hydroxysafflor yellow A, amygdalin, salvianolic acid B, and astragaloside IV (31, 32), all of which have the pharmacological effects of anti-inflammation, anti-oxidation, and renoprotection against diabetes. Astragaloside IV in Radix Astragali (Huangqi) exhibits anti-inflammatory, anti-oxidative stress, and anti-fibrotic properties, effectively alleviating the renal injury induced by high glucose (33, 34). Ferulic acid is known as an anti-oxidative agent, which relieves inflammation, decreases oxidative stress markers, and attenuates excessive autophagy to protect podocytes against injury (35, 36). Astragaloside IV and ferulic acid have also been found to have hypoglycemic effects. Paeoniflorin and hydroxysafflor yellow A alleviate renal injury mainly through inhibiting inflammatory responses (37–39). Amygdalin, hydroxysafflor yellow A, and Salvianolic acid B could ameliorate renal fibrosis by inhibiting the expression of transforming growth factor (TGF)−β1 and the proliferation of renal interstitial fibroblasts (40, 41). The pharmacological effects of these components suggest that the renal protective efficacy of NXT may be a direct effect on the kidney or an indirect anti-diabetic effect.

Collectively, our results provided clinical evidence for the treatment of DKD with NXT capsules. This study was a good attempt for the real-world research of traditional Chinese medicine, which not only proved its feasibility, but also showed the scientific nature. It will serve as a demonstration for the development, marketing and indication expansion of Chinese patent medicines in the future.

However, there are still some limitations in our current study. Firstly, as a retrospective study, although many important confounders were accounted for, residual unknown or unmeasured confounding factors cannot be eliminated. Secondly, the medication data of participants was collected only from hospital records, which may be incomplete because some patients bought drugs at pharmacies. Therefore, the current findings will need to be confirmed in large cohort studies and RCTs.

## Conclusion

NXT can significantly slow the decline of eGFR and reduce the risk of renal outcomes. However, large cohort studies and RCTs are needed to further confirm our results.

## Data availability statement

The original contributions presented in the study are included in the article/**Supplementary Material**. Further inquiries can be directed to the corresponding authors.

## Author contributions

YQZ: Conceptualization, Methodology, Writing - Original Draft; YHZ: Data curation, Investigation; CQY: Software, Validation; YYD: Writing-Reviewing and Editing. LLJ: Writing- Reviewing and Editing; DJ: Data curation, Validation; FML: Methodology, Supervision, Project administration; XLT: Supervision, Project administration. All data were generatedin-house, and no paper mill was used. All authors agree to be accountable for all aspects of work ensuring integrity and accuracy. All authors contributed to the article and approved the submitted version.

## Funding

This work was funded by the 2015 Traditional Chinese Medicine Scientific Research (No. 201507001-11) and the Special Research Funds for Traditional Chinese Medicine Industry from State Administration of Traditional Chinese Medicine (2016ZX03).

## Acknowledgments

The authors thank the statistical assistance of the Tianjin Healthcare and Medical Big Data Platform for the study design, monitoring, and data analysis. The authors also acknowledge the China Academy of Chinese Medical Sciences for supporting the study, and we appreciate the involvement of all authors in this study.

## Conflict of interest

The authors declare that the research was conducted in the absence of any commercial or financial relationships that could be construed as a potential conflict of interest.

## Publisher’s note

All claims expressed in this article are solely those of the authors and do not necessarily represent those of their affiliated organizations, or those of the publisher, the editors and the reviewers. Any product that may be evaluated in this article, or claim that may be made by its manufacturer, is not guaranteed or endorsed by the publisher.
