# Supplementary material for: Naoxintong capsule delay the progression of diabetic kidney disease: A real-world cohort study
Source: Front Endocrinol (Lausanne). 2022 Nov 3;13:1037564. doi: 10.3389/fendo.2022.1037564 (PMC9686849; doi:10.3389/fendo.2022.1037564)
Supplement: Supplementary file 1 [file DataSheet_1.docx]

**Endpoint events:**

1. Doubling of serum creatinine (An increase in creatinine of more than double from baseline);

2. Enter the stage of renal replacement therapy (hemodialysis, peritoneal dialysis, or kidney transplantation);

3. Progress to end-stage renal disease (eGFR＜15 mL/ (min·1.73m2));

4. Die from kidney disease;

5. Major adverse cardiovascular events (MACE) (cardiovascular death, nonfatal myocardial infarction, stroke);

6. Hospitalization for heart failure

**Grouping criteria:**

1. High exposed group: patients taking NXT capsule for 360 days or more;

2. Medium exposed group: patients taking NXT capsules for 180 (inclusive) to 360 (exclusive) days;

3. Low exposed group: patients taking NXT capsules for 60 (inclusive) to 180 (exclusive) days;

4. Non-exposed group: patients without medication records of NXT capsules and did not take traditional Chinese medicine that with the same composition as NXT capsules.

**Study design and screening process**

The study starting point was defined as the date of the first creatinine measurement (meeting inclusion criteria). And study endpoints were defined as the date of the last creatinine measurement or the date of the end point event, whichever was the latter. The scope of the study was defined as the time period between the starting point and the endpoint of the study. The baseline period was defined as the time range from one month before to one month after the study starting point. The outcome period was defined as the time range from one month before to one month after the study endpoint.

According to the inclusion criteria, 298,537 patients who met either of the DKD diagnoses were screened between January 1, 2011 and March 31, 2021. Among those patients who had a creatinine recorded, estimated glomerular filtration rate (eGFR) was calculated. Patients with any eGFR < 90 mL/ min·1.73m^2^ were enrolled in the study. And their first eGFR record (less than 90 mL/ min·1.73m^2^) was defined as baseline eGFR.

In addition to the baseline eGFR, patients needed to have at least one other creatinine record or an endpoint event to reflect treatment effect. After this procedure, 91,695 patients were retained (85,688 of whom had a record of serum creatinine; 13,102 patients with doubled serum creatinine; 3,137 patients underwent renal replacement therapy; 8,012 patients with end-stage renal disease; 272 deaths due to kidney disease; 6,452 hospitalizations for heart failure; 17,131 patients with cardiovascular adverse events).

The cumulative medication time of each patient during the study period was calculated by adding up each prescription period and subtracting the portion beyond the start and endpoints of the study. Then patients were grouped according to grouping criteria. For the exposed group, patients with a medication interval of more than 1 year were excluded (N=449). 1,471 patients were eventually included in the exposed group (490 in high exposed group, 398 in medium exposed group, 583 in low exposed group). For the control group, patients who had used traditional Chinese medicine with the same composition as NXT were excluded from the study (N=81,038), and 8,234 patients were eventually included.

Since the data in this study are real-world data, it is necessary to compare the baseline information of patients in different groups to determine whether there is confounding factors. If there are statistically different variables in the baseline information between the exposed group and non-exposed group, it indicates that patient information does not match between the groups and further processing is required. The propensity score matching (PSM) method was employed to deal with the potential confounding factors between exposed and non-exposed group. After PSM, the control group matched with the high, medium, and low exposed group was defined as non-exposed group 1, non-exposed group 2, and non-exposed group 3, respectively. Through PSM, the three subgroups were matched with corresponding control groups. And all variables were balanced between exposed group and control group. A total of 1,798 patients were enrolled in our final cohort, with 899 patients in each group.

The basic information of patients including demographic characteristics, course of the disease, comorbidities, and baseline medication were collected during the baseline period. The laboratory indices closest to the study starting point were used as the baseline indices. Indicators that are absent during the baseline period will be labeled missing. In the same way, the indicators closest to the study endpoint were used as the outcome indices. Indicators that are absent during the outcome period will be labeled missing.
