# Supplementary material for: Naoxintong capsule delay the progression of diabetic kidney disease: A real-world cohort study
Source: Front Endocrinol (Lausanne). 2022 Nov 3;13:1037564. doi: 10.3389/fendo.2022.1037564 (PMC9686849; doi:10.3389/fendo.2022.1037564)
Supplement: Supplementary file 2 [file DataSheet_2.docx]

| Group | High exposed group (306) | Non-exposed group 1 (306) | P Value | Std. Mean Diff. |
| --- | --- | --- | --- | --- |
| Age (mean (SD)) | 65.74(9.27) | 65.01(10.45) | 0.362 | 0.078 |
| Male (%) | 194(63.4) | 199(65.0) | 0.736 | -0.0347 |
| Smoking history (%) | 60(19.6) | 64(20.9) | 0.532 | -0.0288 |
| Drinking history (%) | 48(15.7) | 50(16.3) | 0.596 | -0.016 |
| BMI (%) |  |  | 0.862 |  |
| obese | 42(13.7) | 46(15.0) |  | -0.0391 |
| overweight | 68(22.2) | 65(21.2) |  | 0.0235 |
| normal | 65(21.2) | 63(20.6) |  | 0.0156 |
| underweight | 1(0.3) | 0(0.0) |  | 0.0513 |
| Blood pressure classification (%) |  |  | 0.93 |  |
| Grade 1 hypertension | 37(12.1) | 36(11.8) |  | 0.0093 |
| Grade 2 hypertension | 13(4.2) | 16(5.2) |  | -0.0464 |
| Grade 3 hypertension | 3(1.0) | 4(1.3) |  | -0.0275 |
| High normal | 142(46.4) | 130(42.5) |  | 0.0787 |
| Normal | 9(2.9) | 10(3.3) |  | -0.0184 |
| Baseline eGFR (mean (SD)) | 71.60 (18.92) | 71.75 (18.59) | 0.921 | -0.0083 |
| Observation days (mean (SD)) | 1546.4 (956.74) | 1570.37 (1021.74) | 0.765 | -0.0246 |
| Baseline medication | | | | |
| Insulin and its analogs (%) | 236(77.1) | 240(78.4) | 0.771 | -0.0347 |
| Glinides (%) | 111(36.3) | 119(38.9) | 0.559 | -0.0524 |
| Sulfonylurea (%) | 87(28.4) | 89(29.1) | 0.929 | -0.0137 |
| Biguanides (%) | 164(53.6) | 160(52.3) | 0.808 | 0.0269 |
| Thiazide diuretics (%) | 20(6.5) | 21(6.9) | 1 | -0.0107 |
| β-blockers (%) | 88(28.8) | 98(32.0) | 0.429 | -0.0708 |
| ACEI (%) | 207(67.6) | 203(66.3) | 0.796 | 0.0316 |
| ARB (%) | 41(13.4) | 47(15.4) | 0.565 | -0.0476 |
| Underlying diseases | | | | |
| Hypertension (%) | 304(99.3) | 303(99.0) | 1 | 0.0513 |
| Cardio-cerebrovascular diseases (%) | 305(99.7) | 305(99.7) | 1 | 0 |
| Chronic respiratory diseases (%) | 70(22.9) | 70(22.9) | 1 | 0 |
| Metabolic diseases (%) | 227(74.2) | 230(75.2) | 0.853 | -0.0253 |
| Urinary system diseases (%) | 80(26.1) | 77(25.2) | 0.853 | 0.0219 |
| Anemia (%) | 69(22.5) | 69(22.5) | 1 | 0 |

Table I The baseline characteristics of high exposed group after PSM.

Table II The baseline characteristics of medium exposed group after PSM.

| Group | Medium exposed group (233) | Non-exposed group 2 (233) | P Value | Std. Mean Diff. |
| --- | --- | --- | --- | --- |
| Age (mean (SD)) | 65.83(8.91) | 66.35(10.02) | 0.558 | -0.0566 |
| Male (%) | 142(60.9) | 126(54.1) | 0.16 | 0.1419 |
| Smoking history (%) | 43(18.5) | 44(18.9) | 0.958 | -0.0103 |
| Drinking history (%) | 36(15.5) | 32(13.7) | 0.714 | 0.0453 |
| BMI (%) |  |  | 0.966 |  |
| obese | 34(14.6) | 34(14.6) |  | 0 |
| overweight | 56(24.0) | 57(24.5) |  | -0.0095 |
| normal | 47(20.2) | 50(21.5) |  | -0.0314 |
| underweight | 1(0.4) | 2(0.9) |  | -0.0607 |
| Blood pressure classification (%) |  |  | 0.936 |  |
| Grade 1 hypertension | 31(13.3) | 34(14.6) |  | -0.0362 |
| Grade 2 hypertension | 11(4.7) | 9(3.9) |  | 0.0413 |
| Grade 3 hypertension | 3(1.3) | 2(0.9) |  | 0.0385 |
| High normal | 103(44.2) | 111(47.6) |  | -0.0687 |
| Normal | 11(4.7) | 9(3.9) |  | 0.0437 |
| Baseline eGFR (mean (SD)) | 71.71 (19.77) | 70.47 (20.49) | 0.508 | 0.0698 |
| Observation days (mean (SD)) | 1297.74 (938.09) | 1333.39 (1020.79) | 0.695 | -0.0354 |
| Baseline medication | | | | |
| Insulin and its analogs (%) | 147(63.1) | 146(62.7) | 1 | 0.01 |
| Glinides (%) | 77(33.0) | 77(33.0) | 1 | 0 |
| Sulfonylurea (%) | 47(20.2) | 50(21.5) | 0.819 | -0.0287 |
| Biguanides (%) | 100(42.9) | 113(48.5) | 0.264 | -0.1121 |
| Thiazide diuretics (%) | 10(4.3) | 12(5.2) | 0.827 | -0.0277 |
| β-blockers (%) | 69(29.6) | 63(27.0) | 0.607 | 0.0559 |
| ACEI (%) | 125(53.6) | 119(51.1) | 0.643 | 0.0568 |
| ARB (%) | 29(12.4) | 30(12.9) | 1 | -0.0109 |
| Underlying diseases | | | | |
| Hypertension (%) | 231(99.1) | 229(98.3) | 0.681 | 0.1214 |
| Cardio-cerebrovascular diseases (%) | 233(100.0) | 233(100.0) | 1 | 0 |
| Chronic respiratory diseases (%) | 52(22.3) | 52(22.3) | 1 | 0 |
| Metabolic diseases (%) | 156(67.0) | 157(67.4) | 1 | -0.0105 |
| Urinary system diseases (%) | 50(21.5) | 48(20.6) | 0.909 | 0.0203 |
| Anemia (%) | 45(19.3) | 49(21.0) | 0.729 | -0.0424 |

Table III The baseline characteristics of low exposed group after PSM.

| Group | Low exposed group (360) | Non-exposed group 3 (360) | P Value | Std. Mean Diff. |
| --- | --- | --- | --- | --- |
| Age (mean (SD)) | 66.76(9.24) | 66.94(10.50) | 0.812 | -0.0193 |
| Male (%) | 219(60.8) | 206(57.2) | 0.363 | 0.0743 |
| Smoking history (%) | 60(16.7) | 69(19.2) | 0.669 | -0.0596 |
| Drinking history (%) | 51(14.2) | 55(15.3) | 0.742 | -0.0296 |
| BMI (%) |  |  | 0.807 |  |
| obese | 52(14.4) | 48(13.3) |  | 0.0325 |
| overweight | 87(24.2) | 89(24.7) |  | -0.0129 |
| normal | 70(19.4) | 64(17.8) |  | 0.042 |
| underweight | 0(0.0) | 1(0.3) |  | -0.0671 |
| Blood pressure classification (%) |  |  | 0.808 |  |
| Grade 1 hypertension | 45(12.5) | 46(12.8) |  | -0.0081 |
| Grade 2 hypertension | 23(6.4) | 28(7.8) |  | -0.0556 |
| Grade 3 hypertension | 8(2.2) | 4(1.1) |  | 0.0817 |
| High normal | 150(41.7) | 142(39.4) |  | 0.0449 |
| Normal | 17(4.7) | 16(4.4) |  | 0.0137 |
| Baseline eGFR (mean (SD)) | 72.99 (17.25) | 73.17 (18.25) | 0.89 | -0.0109 |
| Observation days (mean (SD)) | 1276.47 (1051.08) | 1280.42 (1024.08) | 0.959 | -0.0036 |
| Baseline medication | | | | |
| Insulin and its analogs (%) | 225(62.5) | 228(63.3) | 0.877 | -0.0187 |
| Glinides (%) | 105(29.2) | 105(29.2) | 1 | 0 |
| Sulfonylurea (%) | 78(21.7) | 74(20.6) | 0.784 | 0.0245 |
| Biguanides (%) | 162(45.0) | 148(41.1) | 0.328 | 0.0781 |
| Thiazide diuretics (%) | 17(4.7) | 17(4.7) | 1 | 0 |
| β-blockers (%) | 99(27.5) | 102(28.3) | 0.868 | -0.0179 |
| ACEI (%) | 152(42.2) | 161(44.7) | 0.548 | -0.0515 |
| ARB (%) | 28(7.8) | 20(5.6) | 0.296 | 0.0612 |
| Underlying diseases | | | | |
| Hypertension (%) | 354(98.3) | 356(98.9) | 0.75 | -0.055 |
| Cardio-cerebrovascular diseases (%) | 358(99.4) | 355(98.6) | 0.447 | 0.1425 |
| Chronic respiratory diseases (%) | 67(18.6) | 72(20.0) | 0.706 | -0.0324 |
| Metabolic diseases (%) | 224(62.2) | 223(61.9) | 1 | 0.0063 |
| Urinary system diseases (%) | 77(21.4) | 73(20.3) | 0.783 | 0.0254 |
| Anemia (%) | 70(19.4) | 79(21.9) | 0.462 | -0.0616 |

Table Ⅳ Duration of NXT capsules administration in the exposed group

|  | Number of patients | Mean (days) | Median (days) |
| --- | --- | --- | --- |
| High exposed group | 306 | 700.28 | 592.5 |
| Medium exposed group | 233 | 253.20 | 248 |
| Low exposed group | 360 | 106.82 | 98 |
| Total | 899 | 346.76 | 224 |

Table V The change of eGFR before and after study in patients with high, medium, and low exposure.

|  | High exposed group | Control group 1 | W statistic | P-Value |
| --- | --- | --- | --- | --- |
| Baseline eGFR | 71.6±18.92 | 71.75±18.59 | 46509 | 0.887825 |
| Endpoint eGFR | 71.06±28.37 | 65.97±26.05 | 39958.5 | 0.001712 |
| eGFR change | -0.54±23.76 | -5.78±19.26 | 39567 | 0.000916 |

|  | Medium exposed group | Control group 2 | W statistic | P-Value |
| --- | --- | --- | --- | --- |
| Baseline eGFR | 71.71±19.77 | 70.47±20.49 | 26083 | 0.465422 |
| Endpoint eGFR | 70.79±28.65 | 66.83±25.82 | 23364 | 0.009307 |
| eGFR change | -0.92±21.23 | -3.64±18.2 | 23965 | 0.028736 |

|  | Low exposed group | Control group 3 | W statistic | P-Value |
| --- | --- | --- | --- | --- |
| Baseline eGFR | 72.99±17.25 | 73.17±18.25 | 66672.5 | 0.502315 |
| Endpoint eGFR | 70.41±26.51 | 65.92±27.2 | 57824.5 | 0.012435 |
| eGFR change | -2.58±20.77 | -7.25±21.13 | 56853 | 0.004403 |

Table VI The proportion of changes in CKD stages

|  | Improvement | maintenance | progression | total |
| --- | --- | --- | --- | --- |
| High exposed group | 114 (37.25%) | 127 (41.50%) | 65 (21.24%) | 306 |
| Non-exposed group 1 | 69 (22.55%) | 160 (52.29%) | 77 (25.16%) | 306 |
| Medium exposed group | 79 (33.91%) | 103 (44.21%) | 51 (21.89%) | 233 |
| Non-exposed group 2 | 50 (21.46%) | 131 (56.22%) | 52 (22.32%) | 233 |
| Low exposed group | 113 (31.39%) | 172 (47.78%) | 75 (20.83%) | 360 |
| Non-exposed group 3 | 82 (22.78%) | 183 (50.83%) | 95 (26.39%) | 360 |
